# ^68^Ga-NOTA-CHSg and ^99m^Tc-CHSg Labeled Microspheres for Lung Perfusion and Liver Radiomicrospheres Therapy Planning

**DOI:** 10.1155/2013/279872

**Published:** 2013-12-30

**Authors:** Alejandro Amor-Coarasa, Andrew Milera, Denny Carvajal, Seza Gulec, Jared Leichner, Anthony J. McGoron

**Affiliations:** ^1^Biomedical Engineering Department, Florida International University, 10555 West Flagler Street, EC 2614, Miami, FL 33174, USA; ^2^Herbert Wertheim College of Medicine, Florida International University, 1240 SW 108 Avenue, Path, University Park, FL 33174, USA; ^3^Mount Sinai Medical Center, 4300 Alton Road, Miami Beach, FL 33140, USA; ^4^Jackson North Medical Center, 160 NW 170th Street, North Miami Beach, FL 33169, USA

## Abstract

Fast biodegradable (12 h < half-life < 48 h) radioactive labeled microspheres are needed for PET and SPECT lung perfusion and radiomicrosphere therapy planning. An emulsion method was used to create 30.1 ±4.8 **μ**m size range microspheres with biodegradable Chitosan glycol (CHSg). Microspheres were characterized and labeled with ^99m^Tc or ^68^Ga as an alternative to MAA in perfusion PET and SPECT studies. Surface decoration of CHSg microspheres with p-SCN-Bn-NOTA was performed to increase ^68^Ga  *in vivo* stability. ^99m^Tc was labeled directly to the CHSg microspheres. Labeling yield and *in vitro* radiochemical stability were evaluated. *In vitro* CHSg microsphere degradation half-life was ~24 hours in porcine blood. Labeled microspheres were injected into Sprague Dawley rats and biodistribution was determined after 2 and 4 hours. Both ^99m^Tc-CHSg and ^68^Ga-NOTA-CHSg were quickly allocated in the lungs after injection. ^99m^Tc-CHSg showed 91.6 ± 6.5% and 83.2 ± 4.1% of the decay corrected injected activity remaining in the lungs after 2 and 4 hours, respectively. For the obtained ^68^Ga-NOTA-CHSg microspheres, lung allocation was very high with 98.9 ± 0.2% and 95.6 ± 0.9% after 2 and 4 hours, respectively. The addition of p-SCN-Bn-NOTA acts as a radioprotectant eliminating the released ^68^Ga activity from the lungs to the bladder protecting the other organs.

## 1. Introduction

Since 1974, the use of ^99m^Tc-MAA (macroaggregated albumin) has been established as the gold standard for lung perfusion studies [1]. The availability of a MAA lyophilized kit [1] and the ^99^Mo/^99m^Tc radio-isotopic generator [2] facilitated the use of ^99m^Tc-MAA as a lung perfusion agent. The orientation of macroaggregates (which are seldom spherical) in the blood flow stream is important for determining “effective size,” making it difficult to predict their *in vivo* behavior. Aggregates rupturing into smaller pieces add another factor making the size distribution variable and unreliable [3]. The ideal (theoretical) perfusion particle should be spherical (size not to be dependent on particle orientation) with a practical size distribution of 30 ± 10 *μ*m [3]. Polymeric spherical microparticles with narrow size distributions have been previously obtained [4]. The use of polymerical microspheres will also eliminate the risk of disease transmission due to human derived materials (such as MAA).

Another important application of ^99m^Tc-MAA is in the radiomicrosphere therapy (RMT) planning [5]. Nonspherical macroaggregates (MAA, 10–90 *μ*m) are used to predict the distribution behavior of the perfectly spherical therapy particles used for RMT which are commercially available (SirTEX and TheraSpheres *≈*30 ± 10 *μ*m). Despite the differences in size and morphology, MAA prediction of particle allocation has been shown to be a valuable tool in RMT planning; however, whether a better planning agent can ultimately produce a better clinical outcome is still an open question.

Many of the available biocompatible polymers have been labeled with ^99m^Tc. Some examples are ^99m^Tc-PLGA (poly (DL-lactide-co-glycolide)) nanoparticles [6], ^99m^Tc-PLA (poly lactic acid) [7], and ^99m^Tc-CHS (Chitosan) [8] among others. PLGA and PLA have known long degradation times (months). CHS has been found to have an *in vitro* half-life greater than 21 days [9]. This characteristic is relatively undesired when performing lung perfusion studies or for RMT planning, as the particles need to degrade fast (maximum 48 hour half-life) and restore normal blood flow.

A chitin derivate polysaccharide, chitosan (CHS), has recently emerged as a good material for drug delivery applications [10]. There are several commercially available CHS materials with both high and low solubility in water [11]. Particle degradation half-life is directly related to solubility of the polymer. There is a compromise in the ideal particle degradation rate: it has to be slow enough to allow allocation in the lungs and subsequent imaging, but fast enough to clear the vessels and restore blood flow afterwards (half-life 12–48 hours). One of the best candidates is Chitosan glycol (CHSg). CHSg has moderate (2 mg/mL) solubility in water, is well characterized, and commercially available (Sigma-Aldrich, CHSg ≥ 60%). Degradation can be manipulated using glutaraldehyde as a crosslinking agent.

Another important limitation of RMT planning is the difficulty quantifying biodistribution for dosimetric calculations when ^99m^Tc is used. This is due to the fact that the ^99m^Tc-MAA scintigraphy is a single photon emission tomography technique (SPECT) and creates difficulty in obtaining proper attenuation correction as compared to positron emission tomography (PET). The RMT planning will be greatly benefited by the inclusion of a positron emitter radioisotope, since absolute attenuation correction and potentially superior spatial and temporal resolution are available with PET. Important advances were made in early years by labeling the Pulmolite MAA kit with ^68^Ga [12–14], obtaining an 80% labeling yield and purity >95%. However since MAA is a poor surrogate of therapeutic microspheres, there is a need for fast biodegradable (12 h < half-life < 48 h) microspheres (30 ± 10 *μ*m) that can be labeled with a PET isotope for proper RMT planning.

## 2. Materials and Methods

### 2.1. Particle Preparation and Surface Decoration

CHSg (Sigma-Aldrich, USA) particles were prepared using the water in oil (w/o) emulsion technique. One mL of CHSg solution (2% w/v solution in 2% v/v Acetic Acid) was added dropwise to a round bottom flask containing an egg-shaped magnetic stirrer, 20 mL of toluene, and 100 *μ*L of Tween 80 (surfactant). Magnetic stirring rate was set at 1150 rpm (Model PC-410D, Corning-Cole Palmer, USA). The emulsion was stabilized for 15 minutes and 100 *μ*L of glutaraldehyde (FisherSci, USA) was added. Stirring was continued for another 105 minutes. Later, toluene was decanted. Particles were washed three times with 200-proof ethanol (Sigma-Aldrich, USA) and centrifuged (1000 rcf, 30 seconds); the supernatant was removed and finally lyophilized for labeling (Model Freeze One Plus 6, LabConco, USA).

**Figure 1:**
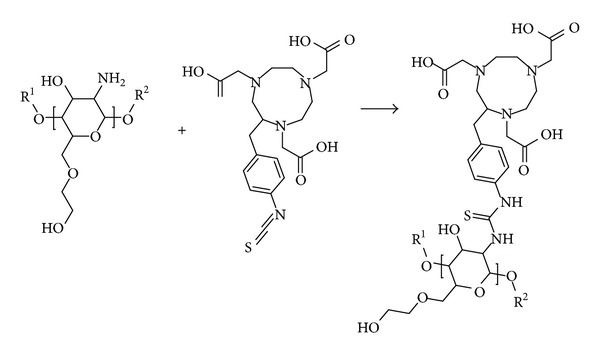
Surface decoration of CHSg microparticles with p-SCN-Bn-NOTA. R^1^ and R^2^ are repeated CHSg polymer chains (equal or different lengths).

A stock solution of p-SCN-Bn-NOTA (Macrocyclics, USA) with concentration 1 mg/mL in Na_2_HCO_3_/NaH_2_CO_3_ (pH 9.3-9.4, Sigma-Aldrich, USA) was prepared. Microspheres were resuspended in 1 mL of the p-SCN-Bn-NOTA Stock solution. The suspension was stirred at room temperature for 4, 12, 24 and 48 hours (*n* = 3 per time point) to form the NOTA-CHSg particles [15]. After p-SCN-Bn-NOTA reaction, particles were washed 3 times with 200-proof ethanol and centrifuged (1000 rcf, 30 seconds); the supernatant was removed and finally lyophilized for labeling (Model Freeze One Plus 6, LabConco, USA). The reaction (Figure 1) yield was evaluated using a UV/Visible spectrophotometer (Cary 100 Bio, Varian/Agilent Technologies, Switzerland) at the 224 nm absorption peak of the p-SCN-Bn-NOTA.

### 2.2. ^99m^Tc-CHSg ^68^Ga-CHSg and ^68^Ga-NOTA-CHSg Labeling

NA^99m^TcO_4_ was obtained from a local pharmacy (Triad Isotopes, Miami, USA). One mCi was used to label approximately 100,000 particles in a 15 mL centrifuge vial containing the lyophilized particles after addition of 100 *μ*L of 1 mg/mL SnCl_2_ stock solution (Sigma-Aldrich, USA). ^99m^Tc-CHSg labeling was performed during 30 minutes at 25°C and 750 rpm in a Thermomixer (Comfort 15 mL Block Thermomixer, Eppendorf, Germany). ^68^Ga was obtained by eluting the IGG-100 (Eckert and Ziegler, 50 mCi) ^68^Ge/^68^Ga generator with 5 mL of 0.1 M HCl solution (starting solution for buffering). ^68^Ga Labeling of ^68^Ga-NOTA-CHSg (pH *≈* 4, 0.25 mL of 3 N NaAc added to initial solution) and ^68^Ga-CHSg (pH *≈* 5.5, 0.3 mL of 3 N NaAc added to initial solution) was performed at room temperature for 15 minutes. Particles were later centrifuged (1000 rcf, 30 seconds) and the supernatant was removed using a 5′′ spinal needle. Both particles and supernatant were measured for labeling yield determination and radiochemical purity studies.

Labeled particles (*n* = 4) were resuspended in reconstituted (1% w/v) bovine hemoglobin lysate (Sigma-Aldrich, USA). Particles were stirred for 4 hours at 37°C in a thermomixer. Every hour the particles were centrifuged, decanted, and measured together with the supernatant to assess radiochemical stability.

### 2.3. CHSg Microspheres Degradation Studies

For the *in vitro* particle degradation studies, porcine blood was obtained from a local abattoir (Mataderos Cabrera, Miami, USA), sodium citrate was added as anticoagulant, and the blood was centrifuged at 3000 rcf for 30 minutes. Plasma was later decanted and used for microsphere degradation experiments. Lyophilized CHSg and NOTA-CHSg (unlabeled) were resuspended in the plasma. CHSg and NOTA-CHSg microsphere samples were extracted at 1, 2, 4, 12, 24, 48, and 72 hours. All samples (*n* = 3 per time point) were analyzed for size distribution and particle concentration using a hemacytometer (Reichert, USA) and an optical microscope (Micromaster, FisherSci, USA).

**Figure 2:**
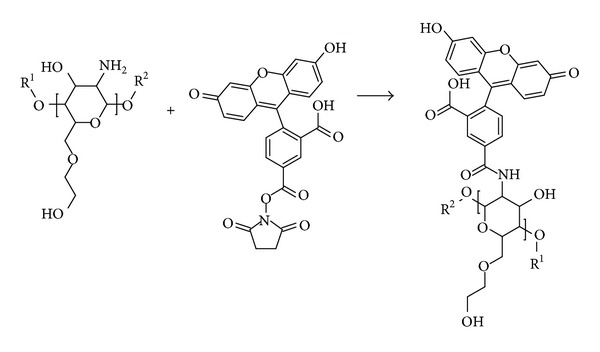
CHSg + NHS-Fluorescein reaction. R^1^ and R^2^ are repeated CHSg polymer chains (equal or different lengths).

For *in vivo* particle degradation studies, lyophilized CHSg microparticles were resuspended in carbonate buffer (pH = 9.34). NHS-Fluorescein (Thermo Scientific, USA) was dissolved in dimethyl sulfoxide (DMSO, Thermo Scientific, USA) with a concentration of 10 mg/mL. A total 1 mg of NHS-fluorescein (100 *μ*L of the stock solution) was added to the vial containing the particles and stirred for 2 hours (Figure 2). At the end of the reaction particles were centrifuged, washed three times with water, and finally lyophilized (lyophilized CHSg-fluorescein).

Particles were resuspended in saline solution for injection (FisherSci, USA) and *≈*10000 particles were injected to each Sprague Dawley rat (200–225 grams, Harlan, USA) in the lateral tail vein. Animals were euthanized at 2, 6, 12, and 24 hours (*n* = 2 per time point). Before extraction of the lungs, the trachea was isolated and a V cut was made. A syringe containing optimal cutting temperature (OCT, Tissue-Tek, USA) cryoembedding media was inserted in the trachea and the lungs were filled with 2 mL. Lungs were finally extracted and frozen in a plastic mold filled with OCT and dipped in liquid nitrogen. Specimens were obtained by cryosectioning the frozen samples (14 *μ*m slices) in a Microtome (Leica, Japan). Lung cryospecimens (4 lung sections per animal) were analyzed with a florescence microscope (Olympus IX81 with a Q Imaging Retiga 1300 Camera, USA). The entire area of each specimen was imaged using a 4x objective. Obtained images were analyzed using in-house software and particles were measured and counted.

### 2.4. Lung Perfusion Experiments

Animals (Sprague Dawley rats, 200–225 grams, 2 per time point, Harlan, USA) were weighed before the procedure and anesthetized using an Ohmeda Isotec 3 isoflurane vaporizer (GE Healthcare, USA). Animals were restrained in the supine position (completely anesthetized) and a torso X-ray was obtained (Belmont Acuray 071A, USA). Later, 100 *μ*L of the labeled microspheres (8,000–10,000 particles) with an activity ranging from 1.85 to 3.7 MBq (50 to 100 *μ*Ci) was injected through the lateral tail vein. Animals were euthanized at 2 or 4 hours. For both time-points lungs, liver, spleen, heart, kidneys, ribs, and 0.2 mL of blood and urine were collected, weighed, and measured for activity using a Cobra 5000 well counter (Packard, USA). Noncollimated autoradiography images (in the unaltered supine position the X-ray was obtained) were also taken at 1, 2, 3, and 4 hours (Packard Phosphorimager, Perkin Elmer, USA). In one group free ^68^Ga was injected as a control. The obtained X-rays and the autoradiography images were superimposed to provide anatomical and functional data.

## 3. Results and Discussion

### 3.1. Particle Preparation and Characterization

**Figure 3:**
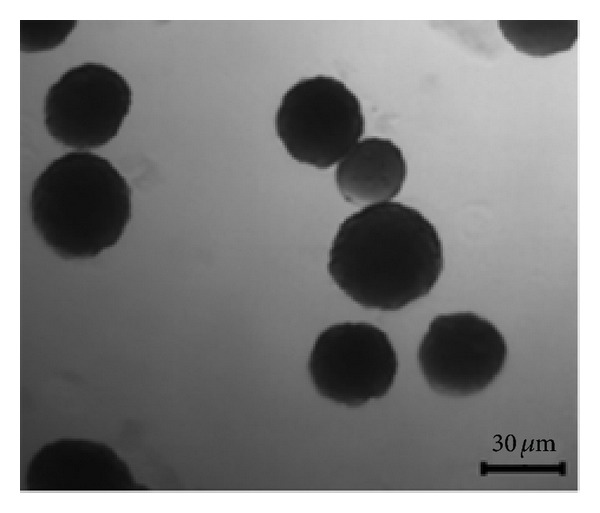
Prepared CHSg microspheres.

The emulsion method used created spherical particles (Figure 3). A size distribution of 30 ± 5 *μ*m was obtained.

Lyophilization and labeling of the particles did not affect their size and morphology. CHSg microspheres swelled 20–25% when placed in contact with water, a feature that was taken into consideration when producing these particles.

**Figure 4:**
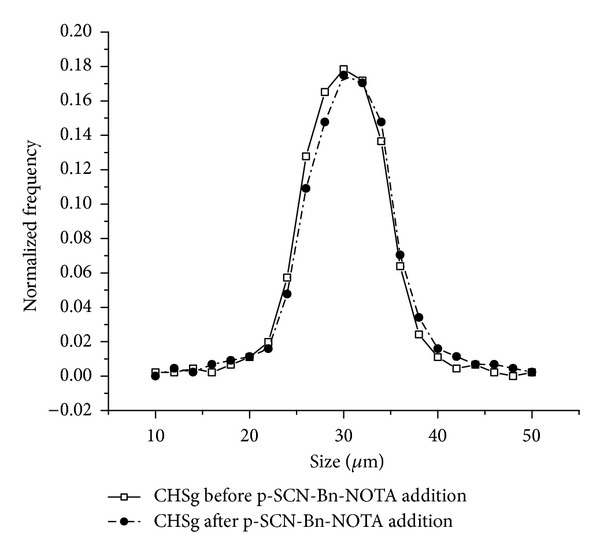
CHSg size distribution before and after p-SCN-Bn-NOTA surface decoration.

The distribution did not change after the 12-hour reaction with p-SCN-Bn-NOTA (Figure 1) (*P* = 0.1, 95% confidence). The final size distribution obtained was 31 ± 5 *μ*m (Figure 4). However many broken particles (no longer spherical) and small fragments were observed due to rupture of the original CHSg microspheres after 24 and 48 hours of p-SCN-Bn-NOTA reaction.

**Figure 5:**
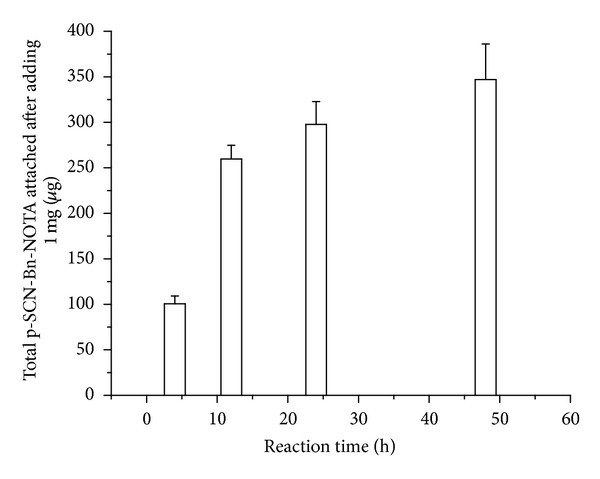
Surface decoration of CHSg with p-SCN-Bn-NOTA reaction yield at different reaction times.

Around 260 ± 15 *μ*g of p-SCN-Bn-NOTA (of the total 1 mg added, *n* = 3) was covalently attached to the surface of the CHSg microspheres after 12 hours of reaction. The net p-SCN-Bn-NOTA amount that bonded to the microspheres surface was increased to 297 ± 25 and 347 ± 40 *μ*g after 24 and 48 hours of reaction (Figure 5).

Maximum reaction yield taking into consideration the totality of available NH_2_ groups was slightly over 1%. Nevertheless it is a skewed calculation since only a fraction of these groups are exposed for the p-SCN-Bn-NOTA reaction. Regardless of the yield, the addition of 260 micrograms of p-SCN-Bn-NOTA to the batch represents a theoretical loading capacity (assuming 95% labeling yield) of 12.8 *μ*Ci/particle (1.28 Ci for 100,000 particles). In molecular imaging only 3–5 mCi is used, 3 orders of magnitude less than the total available capacity.

### 3.2. ^99m^Tc-CHSg ^68^Ga-CHSg and ^68^Ga-NOTA-CHSg Labeling and *In Vitro* Stability

**Figure 6:**
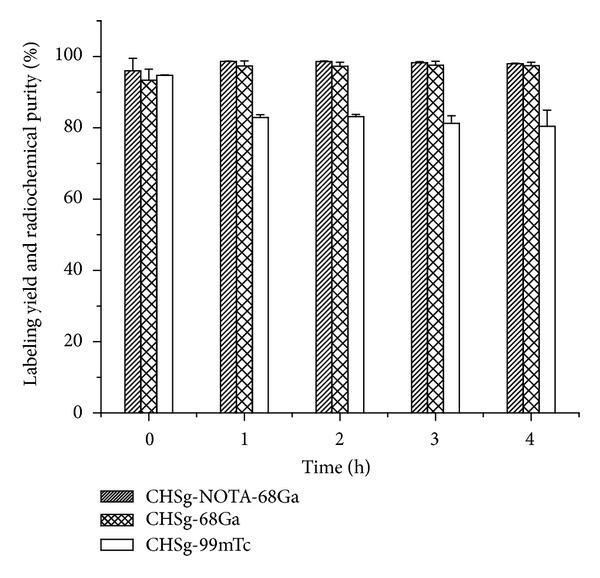
Labeling yield and *in vitro* radiochemical purity of ^99m^Tc-CHSg ^68^Ga-CHSg and ^68^Ga-NOTA-CHSg.

Labeling yields of 94.7 ± 0.1%, 96.0 ± 3.5%, and 97.4 ± 3.0% for ^99m^Tc-CHSg, ^68^Ga-CHSg, and ^68^Ga-NOTA-CHSg, respectively, were obtained (Figure 6). Over 97% radiochemical purity was found at all times for ^68^Ga-CHSg and ^68^Ga-NOTA-CHSg during the 4-hour study. Within the first hour ^99m^Tc-CHSg microparticles quickly decreased in radiochemical purity to 82%, remaining fairly constant afterwards (being 80% after 4 hours) (Figure 6).

Radiochemical purity in saline solution and PBS buffer was over 90% for ^99m^Tc-CHSg, ^68^Ga-CHSg, and ^68^Ga-NOTA-CHSg after four hours. Even though the *in vitro* radiochemical purity of ^99m^Tc-CHSg decreased rapidly to 80% in 1% reconstituted hemoglobin lysate one hour after labeling, the particles were tested in animals. For lung perfusion particles allocated in the vessels, the occlusion locally reduces the fluid in contact with the particles, potentially reducing the detachment of the ^99m^Tc label and therefore increasing the chances of a successful image. Because of the high labeling yield obtained for ^99m^Tc-CHSg (96.1 ± 0.3%) the particles did not need postlabeling purification.

### 3.3. CHSg Microspheres Degradation Studies

**Figure 7:**
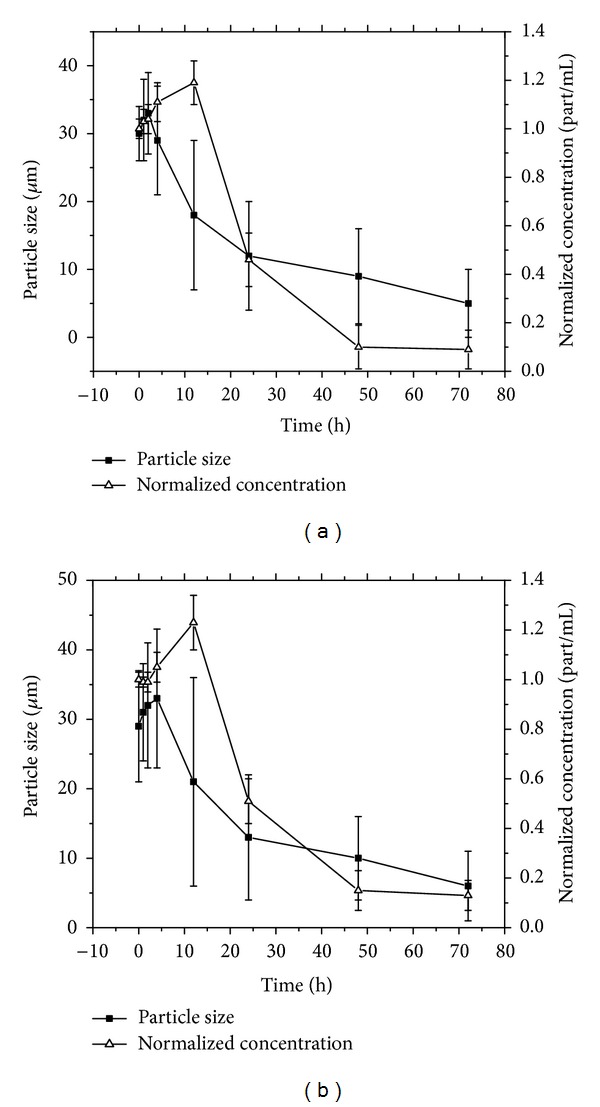
Porcine plasma microsphere degradation studies for (a) CHSg and (b) CHSg-NOTA (*n* = 3).

Porcine plasma studies showed fast (*≈*24 hours) microsphere degradation half-life (Figure 7). CHSg particles are initially swollen in plasma (effect not observed in PBS buffer or saline solution). The dramatic increase observed in particle concentration after 12 hours, together with the decrease in average particle size, is due to the rupture of the original particles into smaller fractions. These fractions are later dissolved (degraded) disappearing gradually from the suspension. The observed half-lives for CHSg and CHSg-NOTA were similar (*≈*24 hours), and there was no significant difference in their degradation profiles.

**Figure 8:**
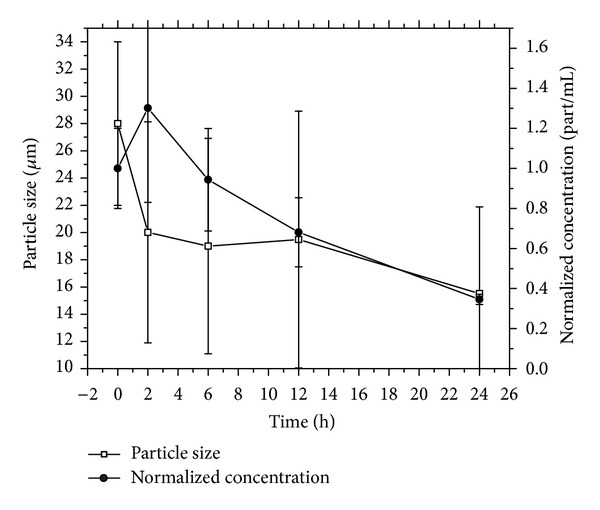
CHSg microspheres *in vivo* degradation studies (*n* = 2 per time point).

Particle degradation experiments performed *in vivo* show a similar degradation mechanism to that obtained *in vitro* (Figure 8).

**Figure 9:**
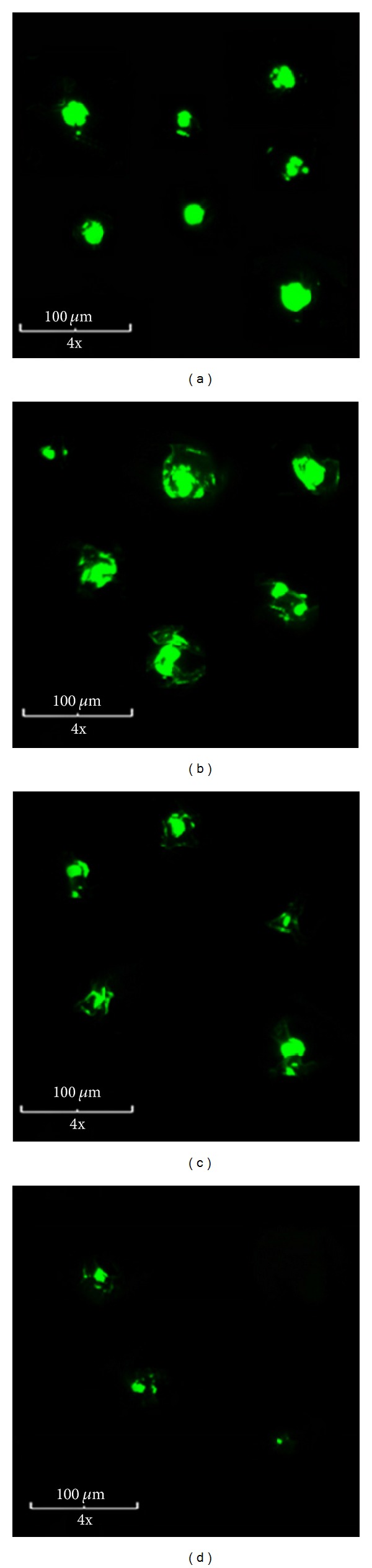
Representation of the *in vivo* CHSg microsphere degradation mechanism. Collage of representative microarticles found in the cryosections for different time points: (a) 2 hours, (b) 6 hours, (c) 12 hours, and (d) 24 hours. Relative particle amount in the images is related to the real particle concentration found in the tissue sections.

Particle concentration is initially increased because of microspheres rupture. Smaller pieces are later slowly dissolved. This dissolution results in a steady decrease of the particles average diameter and also in particle concentration in tissue (Figure 9). The *in vivo* half-life of the particles was determined to be 18–20 hours, since the time when the particle concentration is reduced to half (50% of initial particle concentration of any size occluding lung capillaries).

Results obtained in the *in vivo* experiments are highly qualitative since many assumptions were made for the calculations of average size and concentration. Particles concentration was assumed to be homogeneous in the entire lung, and particles under 10 micrometers were not included in the analysis since those should no longer occlude the vessels and therefore will not interfere with the later injection of the therapeutic particles in the final RMT phase of the procedure.

Furthermore, the average size calculation is only an estimate due to the artifacts inherent in the cryosectioning method; there is no way to know if a particle that is sized represents the whole cross-section or only a part of the particle, the latter being more likely. Therefore, the particle size distribution is probably an underestimate of the true *in vivo* size distribution. Nevertheless, by 24 hours, comparatively few particles were observed in the sections. Also, no particle clumping was observed as the particles were found to be evenly distributed and thus a good estimation of particles half-life was obtained. The particle degradation half-life was found to be between 18 and 20 hours, which makes the CHSg microspheres a potential candidate for lung perfusion imaging and RMT planning.

### 3.4. Lung Perfusion Experiments

Following tail vein injection, most of the ^99m^Tc-CHSg activity was allocated in the lungs within the first 30 seconds (checked with Geiger counter, Victoreen ASM-990, Fluke, USA). After 2 hours, 91.6 ± 6.5% of the injected activity (decay corrected) was allocated in the lungs, and after 4 hours 83.2 ± 4.1% was still found in the lungs, as measured using a gamma-well counter (Cobra 5000 well counter, Packard, USA) (Table 1). The activity released from the lungs was almost exclusively excreted to the urine accounting for 4.9  ±  2.5% and 10.0  ±  2.1% decay corrected injected dose (DC-ID) after 2 hours and 4 hours, respectively. Less than 3% DC-ID/organ was detected in all the other organs at any given time point (Table 1). Lung perfusion images were obtained at several time points confirming the preferential lung allocation (Figure 11).

**Figure 10:**
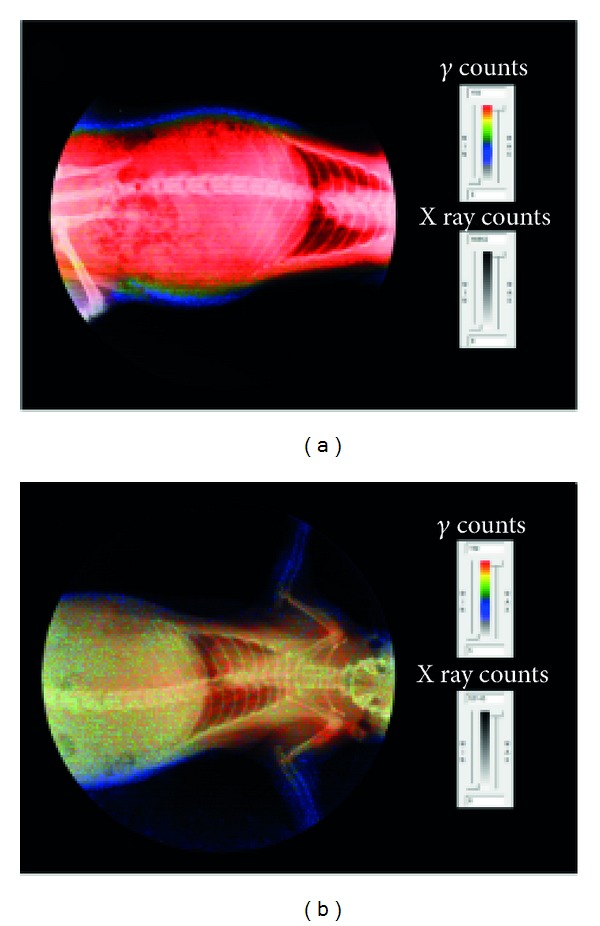
Comparison of images 10 minutes after injection for (a) free ^68^Ga and (b) ^68^Ga-CHSg.

Despite the excellent *in vitro* results obtained, ^68^Ga-CHSg did not perform well as a lung perfusion imaging agent. Strong evidence of ^68^Ga transchelation by native transferrin was observed (Figure 10).

**Figure 11:**
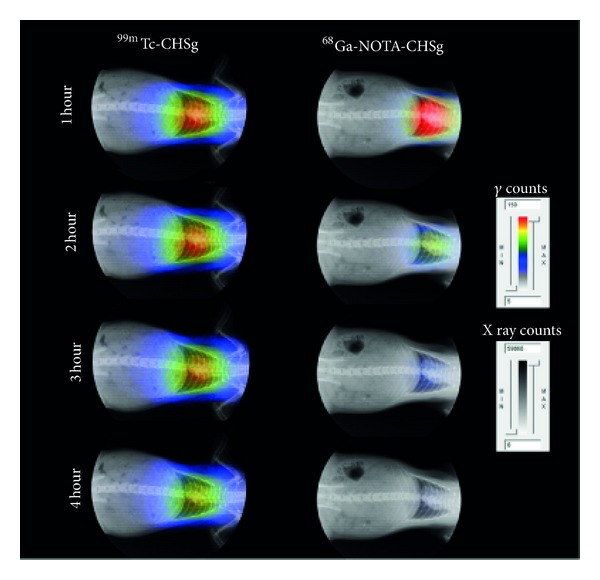
Uncollimated, non-decay-corrected full body X-ray/autoradiography of ^99m^Tc-CHSg and ^68^Ga-NOTA-CHSg at 1, 2, 3, and 4 hours following injection.

Nevertheless, ^68^Ga-CHSg *in vivo* behavior was notably different from that of free ^68^Ga 2 hours after injection. After 2 hours 31.9 ± 1.3% DC-ID from the ^68^Ga-CHSg can be found in the lungs (Table 1), compared to the 3.1 ± 2.9% for the free ^68^Ga, but 46.7 ± 1.2% of the activity from the ^68^Ga-CHSg was already released to the blood. The ^68^Ga release from the CHSg to the blood was not expected since *in vitro* experiments showed minute leaching. This result (release of isotope from ^68^Ga-CHSg) made it necessary to coat the surface of the CHSg microspheres with a ^68^Ga specific chelator (NOTA) to increase *in vivo* stability of the labeling. Animal experiments performed with ^68^Ga-NOTA-CHSg showed high lung allocation and stability during the 4-hour study (Figure 11).

After 2 hours 98.9 ± 0.2% DC-ID of ^68^Ga-NOTA-CHSg was found in the lungs, decreasing to 95.6 ± 0.9% after 4 hours. The activity released from the lungs moved directly to the bladder (3.5 ± 0.6% after 4 hours). The absence of activity in the blood (0.1 ± 0.1% DC-ID at 2 hours and 0.5 ± 0.1% DC-ID after 4 hours) evidenced the high radiochemical stability of the NOTA-^68^Ga complex. The activity found in the urine must be due to early particle degradation releasing small polar fragments as frag-NOTA-^68^Ga. The addition of the NOTA chelator to the surface of the particles also served as an apparent radioprotectant to the rest of the organs, since less than one percent DC-ID was found in the blood at any given time. From the images (Figure 11) and the organ biodistribution data (Table 1), it can be concluded that *in vivo* stability of the prepared ^68^Ga-NOTA-CHSg and ^99m^Tc-CHSg is remarkable and the labeled microspheres are good candidates for PET and SPECT lung perfusion imaging, respectively.

## 4. Conclusions

Chitosan glycol microspheres within the desired 30 ± 10 *μ*m size range (to act as surrogates for the commercial SirSpheres and TheraSpheres, for treatment planning) were successfully obtained. Labeling was performed with >90% yield and *in vitro* radiochemical stability after 4 hours. High *in vitro* radiochemical purity was found for the labeled particles in the 4 hour study. *In vitro* degradation half-life was found to be *≈*24 hours in porcine plasma, while *in vivo* half-life of the particles was found to be 18–24 hours. Both ^99m^Tc-CHSg and ^68^Ga-NOTA-CHSg showed lung allocation quickly after injection. Biodistribution showed 91.6 ± 6.5% DC-ID and 83.2 ± 4.1% DC-ID of the ^99m^Tc-CHSg injected activity remaining in the lungs after 2 and 4 hours, respectively. Lung allocation was very high for ^68^Ga-NOTA-CHSg microspheres, with 98.9 ± 0.2% DC-ID and 95.6 ± 0.9% DC-ID after 2 and 4 hours, respectively. The addition of p-SCN-Bn-NOTA acts as a ^68^Ga radioprotectant, since the microspheres degradation products are quickly eliminated to the bladder protecting the other organs, contrary to free ^68^Ga which remains in the blood pool bound to native transferrin.

## Figures and Tables

**Table 1:** Biodistribution for ^99m^Tc-CHSg and ^68^Ga-NOTA-CHSg at 2 and 4 hours and ^68^Ga-CHsg at 2 hours expressed as percent of decay corrected injected dose per organ (% DC-ID/organ).

Organs	Biodistribution in % DC-ID/organ					
	^ 68^Ga-NOTA-CHSg		^ 99m^Tc-CHSg		^ 68^Ga-CHSg	Free ^68^Ga
	2 hours	4 hours	2 hours	4 hours	2 hours	2 hours
Spleen	0.1 ± 0.0	0.1 ± 0.0	0.0 ± 0.0	0.1 ± 0.0	0.9 ± 0.2	0.6 ± 0.5
Blood	0.1 ± 0.1	0.5 ± 0.1	0.7 ± 0.5	0.8 ± 0.1	46.7 ± 1.2	84.9 ± 4.5
Rib	0.0 ± 0.0	0.0 ± 0.0	0.0 ± 0.0	0.0 ± 0.0	0.4 ± 0.0	0.5 ± 0.4
Urine	0.5 ± 0.4	3.5 ± 0.6	4.9 ± 2.5	10.0 ± 2.1	10.9 ± 0.1	6.8 ± 2.9
Right kidney	0.1 ± 0.0	0.1 ± 0.0	1.1 ± 1.0	2.6 ± 0.8	0.9 ± 0.2	0.5 ± 0.5
Left kidney	0.1 ± 0.0	0.1 ± 0.0	1.2 ± 1.1	2.6 ± 0.8	0.8 ± 0.3	0.5 ± 0.5
Heart	0.0 ± 0.0	0.0 ± 0.0	0.0 ± 0.0	0.0 ± 0.0	1.2 ± 0.2	0.7 ± 0.7
Total lungs	98.9 ± 0.2	95.6 ± 0.9	91.6 ± 6.5	83.2 ± 4.1	31.9 ± 1.3	3.1 ± 2.9
Total liver	0.2 ± 0.1	0.2 ± 0.1	0.3 ± 0.3	0.7 ± 0.3	6.3 ± 0.7	2.3 ± 1.9

Bold highlights the most important target organs. It is necessary to keep most of the activity in the lungs while the blood stays clear.

## Abbreviations

MAA: Macroaggregated albumin
PET: Positron emission tomography
RMT: Radiomicrosphere therapy
SPECT: Single photon emission tomography
CHSg: Chitosan glycol
NOTA: 1,4,7-triazacyclononane-1,4,7-triacetic acid
p-SCN-Bn-NOTA: S-2-(4-Isothiocyanatobenzyl)-1,4,7-triazacyclononane-1,4,7-triacetic acid.
